# On the use of the healthy lifestyle index to investigate specific disease outcomes

**DOI:** 10.1038/s41598-024-66772-w

**Published:** 2024-07-15

**Authors:** Vivian Viallon, Heinz Freisling, Komodo Matta, Anne Østergaard Nannsen, Christina C. Dahm, Anne Tjønneland, Anne Kirstine Eriksen, Rudolf Kaaks, Verena A. Katzke, Matthias B. Schulze, Giovanna Masala, Giovanna Tagliabue, Vittorio Simeon, Rosario Tumino, Lorenzo Milani, Jeroen W. G. Derksen, Yvonne T. van der Schouw, Therese Haugdahl Nøst, Kristin Benjaminsen Borch, Torkjel M. Sandanger, J. Ramón Quirós, Miguel Rodriguez-Barranco, Catalina Bonet, Amaia Aizpurua-Atxega, Lluís Cirera, Marcela Guevara, Björn Sundström, Anna Winkvist, Alicia K. Heath, Marc J. Gunter, Elisabete Weiderpass, Mattias Johansson, Pietro Ferrari

**Affiliations:** 1 International Agency for Research On Cancer (IARC-WHO), Lyon, France; 2 Department of Public Health, Aarhus University, Aarhus, Denmark; 3 Danish Cancer Society Research Center, Copenhagen, Denmark; 4 Department of Public Health, University of Copenhagen, Copenhagen, Denmark; 5 Division of Cancer Epidemiology, German Cancer Research Center (DKFZ), 69120 Heidelberg, Germany; 6 Department of Molecular Epidemiology, German Institute of Human Nutrition Potsdam-Rehbruecke, Nuthetal, Germany; 7 German Center for Diabetes Research (DZD), Neuherberg, Germany; 8 Institute of Nutritional Science, University of Potsdam, Nuthetal, Germany; 9 Institute for Cancer Research, Prevention and Clinical Network (ISPRO), Florence, Italy; 10 Cancer Registry Unit, Fondazione IRCCS Istituto Nazionale Dei Tumori, Milan, Italy; 11 Unit of Medical Statistics, University “L. Vanvitelli”, Naples, Italy; 12 Hyblean Association for Epidemiological Research, AIRE–ONLUS, Ragusa, Italy; 13 Unit of Cancer Epidemiology, Città Della Salute E Della Scienza University-Hospital, and Center for Cancer Prevention (CPO), Turin, Italy; 14 Julius Center for Health Sciences and Primary Care, University Medical Center Utrecht, Utrecht University, Utrecht, The Netherlands; 15 K.G. Jebsen Center for Genetic Epidemiology, Department of Public Health and Nursing, NTNU - Norwegian University of Science and Technology, Trondheim, Norway; 16 Department of Community Medicine, UiT The Arctic University of Norway, Tromsø, Norway; 17 Public Health Directorate, Asturias, Spain; 18 Escuela Andaluza de Salud Pública (EASP), Granada, Spain; 19 Instituto de Investigación Biosanitaria Ibs.GRANADA, Granada, Spain; 20 Present Address: Centro de Investigación Biomédica en Red de Epidemiología y Salud Pública (CIBERESP), Madrid, Spain; 21 Unit of Nutrition and Cancer, Catalan Institute of Oncology - ICO, L’Hospitalet de Llobregat, Barcelona, Spain; 22 Nutrition and Cancer Group; Epidemiology, Public Health, Cancer Prevention and Palliative Care Program, Bellvitge Biomedical Research Institute - IDIBELL, L’Hospitalet de Llobregat, Barcelona, Spain; 23 Sub Directorate for Public Health and Addictions of Gipuzkoa, Ministry of Health of the Basque Government, San Sebastián, Spain; 24 Epidemiology of Chronic and Communicable Diseases Group, Biodonostia Health Research Institute, San Sebastián, Spain; 25 Department of Epidemiology, Murcia Regional Health Council, IMIB-Arrixaca, Murcia, Spain; 26 Instituto de Salud Pública y Laboral de Navarra, 31003 Pamplona, Spain; 27 Navarra Institute for Health Research (IdiSNA), 31008 Pamplona, Spain; 28 Department of Public Health and Clinical Medicine, Umeå University, Umeå, Sweden; 29 Department of Public Health and Clinical Medicine, Sustainable Health, Umeå University, Umeå, Sweden; 30 Department of Internal Medicine and Clinical Nutrition, The Sahlgrenska Academy, University of Gothenburg, Gothenburg, Sweden; 31 Department of Epidemiology and Biostatistics, School of Public Health, Imperial College London, London, UK

**Keywords:** Healthy lifestyle index, Lifestyle factors, Mortality, Type 2 diabetes, Cardiovascular diseases, Cancer, Composite score, Cancer, Diseases, Medical research

## Abstract

The healthy lifestyle index (HLI), defined as the unweighted sum of individual lifestyle components, was used to investigate the combined role of lifestyle factors on health-related outcomes. We introduced weighted outcome-specific versions of the HLI, where individual lifestyle components were weighted according to their associations with disease outcomes. Within the European Prospective Investigation into Cancer and Nutrition (EPIC), we examined the association between the standard and the outcome-specific HLIs and the risk of T2D, CVD, cancer, and all-cause premature mortality. Estimates of the hazard ratios (HRs), the Harrell’s C-index and the population attributable fractions (PAFs) were compared. For T2D, the HR for 1-SD increase of the standard and T2D-specific HLI were 0.66 (95% CI: 0.64, 0.67) and 0.43 (0.42, 0.44), respectively, and the C-index were 0.63 (0.62, 0.64) and 0.72 (0.72, 0.73). Similar, yet less pronounced differences in HR and C-index were observed for standard and outcome-specific estimates for cancer, CVD and all-cause mortality. PAF estimates for mortality before age 80 were 57% (55%, 58%) and 33% (32%, 34%) for standard and mortality-specific HLI, respectively. The use of outcome-specific HLI could improve the assessment of the role of lifestyle factors on disease outcomes, thus enhancing the definition of public health recommendations.

## Introduction

Lifestyle behaviors encompass multiple exposure factors, such as smoking habits, alcohol consumption, physical activity, adiposity, dietary habits and sleep^1,2^. Unhealthy lifestyle behaviors are associated with an increased risk of several chronic diseases^3–9^ and all-cause mortality^10^. This was first established in studies of individual lifestyle components, whereby summary measures such as the Mediterranean Diet Score for diet^11–13^, the number of pack-years for smoking habits^14,15^ and body mass index (BMI) for adiposity^16^, were associated with health-related outcomes. In parallel, individual lifestyle factors have been combined into versions of the healthy lifestyle index (HLI) to study the combined effects of individual lifestyle components on health and provide a holistic assessment on the role of lifestyle^5,6,9,10,17,18^. The HLI was mostly defined as the sum of individual scores expressing exposure to one particular lifestyle component, and was associated with mortality^2,6,19^ and the risk of type 2 diabetes (T2D)^17,20–22^, cardiovascular diseases (CVD)^3,5^, cancers^18,23–28^ and multi-morbidity^8,29^.

Although alternative versions have been proposed, e.g. based on principal component analysis^19^, the standard version of the HLI gives equal weight to each lifestyle component score, which implicitly assumes that all components have the same relationship with a given disease outcome. This strategy could yield biased assessments of the lifestyle-outcome relationships, particularly for outcomes that are predominantly associated with one lifestyle component. In this study we introduced and examined outcome-specific HLIs that used outcome-specific weights reflecting the strength of the association between each component and the outcome.

Within the European Prospective Investigation into Cancer and nutrition (EPIC)^30^, we compared results of analyses based on the standard and the outcome-specific HLIs in relation to the risk of T2D, CVD, overall cancer and all-cause mortality. We focused our evaluation on three standard epidemiological quantities: the hazard-ratio (HR), Harrell’s C-index, and population attributable fractions (PAFs) to reflect the strength of association, the discriminatory power, and the public health burden, respectively. This empirical comparison was complemented with a theoretical study of unweighted and weighted composite scores under simple linear causal models.

## Methods

### Study population

EPIC is an ongoing multicentric prospective study originally designed to study the relationship between diet and cancer risk^30^. EPIC recruited over 500,000 men and women between 1992 and 2000 from 23 centers in 10 European countries. In our analyses we excluded participants from centres lacking information on occurrence or date of diagnosis of T2D or CVD (France, Norway, Greece and Malmö; n = 168,382), participants with no follow-up for mortality (n = 1746) or no information on lifestyle (n = 934), participants with missing information on the incidence of T2D, CVD, and/or cancer during follow-up (n = 63,842), participants with prevalent T2D, CVD, or cancer at recruitment (n = 23,864), and, for sake of simplicity, participants with missing information on any of the five variables used in the definition of HLI (n = 5786), defined as complete-case analysis.

### Health-related outcomes

Data on vital status and incidence of T2D and CVD (coded using the 10th Edition of the International Classification of Diseases, ICD-10), and cancer (coded according to the International Classification of Diseases for Oncology, ICD-O-3) were collected by each participating centre, from inclusion in the study to a center- and outcome-specific last date of ascertainment^30–32^. Dates of death were collected using record linkage with cancer registries, boards of health and death indices, or through active follow-up. Incident T2D cases, defined as E11 (ICD-10), were ascertained by a combination of self-report, linkage to primary care registers, secondary care registers, medication use (drug registers), hospital admissions, and mortality data^31^. CVD endpoints, defined as a composite of ischemic heart diseases (I20-I25), atrial fibrillation (I48), and cerebrovascular disease (I60-I69), were ascertained by different methods depending on the follow-up procedures by centre, using active follow-up through questionnaires or linkage with morbidity and hospital registries, or both^32^. Incident first primary cancer cases (excluding non-melanoma skin cancers) were identified through a combination of center-specific methods, including health insurance records, cancer and pathology registries and active follow-up through study participants and their next-of-kin. Follow-up for each participant and event of interest began upon inclusion in the study and ended upon the occurrence of the event, loss to follow-up, or the last date of ascertainment, whichever came first.

### Assessment of lifestyle exposures at baseline

BMI (kg/m2) was derived from measured height and weight in all centers, except Oxford where it was self-reported^30^. A validated index capturing all physical activity domains (Cambridge Index) was computed from physical activity during recreational activities and at work^33^. Diet, including alcohol intake, was assessed using validated country- or center-specific dietary questionnaires designed to capture habitual consumption over the year preceding the study recruitment^30^. To measure adherence to a healthy diet, we computed the modified relative Mediterranean Diet Score (mrMDS), a version of the original Mediterranean Diet Score incorporating vegetable oil instead of olive oil^12^. To avoid redundancy with the alcohol component in the HLI, our version of mrMDS omitted alcohol intake. The remaining eight mrMDS components were measured in grams per 1000 kcal to express dietary intake as energy density^12^. All dietary components were divided into country-specific tertiles and scores 0 to 2 were summed up, resulting in a final mrMDS ranging from 0 to 16 with increasing scores for healthier diets. Information on smoking status was obtained using lifestyle questionnaires^30^, as was information on variables used for adjustments in our models, including educational attainment, menopausal status in women and the use of hormones in post-menopausal women.

### Healthy lifestyle indices

Following the previous definition of the HLI used in a study of multi-morbidity in EPIC^8^, we considered HLIs that combined information on participants’ exposure to smoking, alcohol intake, diet, physical activity and adiposity. To facilitate the comparison of performance between the standard and outcome-specific HLIs, we used a binary scoring with 0/1 values reflecting unhealthy/healthy behavior for each component^8^, as displayed in Table 1. The standard HLI, ranging from 0 (unhealthiest behavior) to 5 (healthiest behavior), was defined as

$$standard\;HLI = Smoking^{{(0,1)}} + Alcohol^{{(0,1)}} + Diet^{{(0,1)}} + PA^{{(0,1)}} + Adipo^{{(0,1)}} .$$

**Table 1:** Binary and categorical scores used for the computation of the standard and outcome-specific HLIs, following a previous definition of the HLI^8^.

Modifiable lifestyle factor	Binary scores		Categorical scores	
Alcohol intake				
g/d	< 6 (W) or < 12 (M)	1	< 6	4
	≥ 6 (W) or ≥ 12 (M)	0	≥ 6 to < 12	3
			≥ 12 to < 25	2
			≥ 25 to < 60	1
			≥ 60	0
Body mass index				
kg/m^2^	≥ 18.5 to < 30	1	≥ 22 to < 24	4
	< 18.5	0	< 22	3
	≥ 30	0	≥ 24 to < 26	2
			≥ 26 to < 30	1
			≥ 30	0
Mediterranean diet score				
Quantiles	≥ median	1	Q5	4
	< median	0	Q4	3
			Q3	2
			Q2	1
			Q1	0
Physical activity index				
Categories	Active	1	Active	4
	Moderately active	1	Moderately active	3
	Moderately inactive	0	Moderately inactive	1
	Inactive	0	Inactive	0
Smoking status				
Categories	Never	1	Never	4
	Former	1	Former	2
	Current	0	Current	0

W, women; M, men.

To more accurately reflect the potential heterogenous relationships of each component with specific disease outcomes, outcome-specific HLIs were constructed using the same categorical scoring system. Data-driven weights were derived from the parameters of the main effects (${w}_{k}$) and of the interaction terms (${\gamma }_{l}$) in outcome-specific adjusted Cox models, implementing a forward selection procedure in EPIC to select relevant interaction terms among the lifestyle components. The outcome-specific HLI was defined as

$$Outcome-specific HLI{ } = \mathop \sum \limits_{{{\text{k}} = 1}}^{5} w_{k}^{*} *\left( {Summary\; Variable} \right)_{k} + \sum \gamma_{l}^{*} *\left( {Interaction \;Term} \right)_{l}$$

with weights ${w}_{k}^{*}$ and ${\gamma }_{l}^{*}$ corresponding to scaled versions of ${w}_{k}$ and ${\gamma }_{l}$ so that outcome-specific HLIs had unit variance and larger values correspond to healthier profiles.

We also considered a more comprehensive scoring system for each variable ranging from 0 (unhealthiest) to 4 (healthiest behavior), as displayed in Table 1, again following a previous definition of the HLI used in EPIC^8^.

### Cox models

In all our analyses, Cox models used age as the main time scale and were stratified by study center, sex, and age at recruitment in 5-year categories. They were adjusted for education level (no schooling, primary, secondary, and university or more), height (continuous), and energy intake from non-alcoholic sources (kcal/day), and, for women, menopausal status (pre-menopausal, peri-menopausal, post-menopausal, surgical) and use of postmenopausal hormones (never, ever, unknown). For each outcome, one Cox model was constructed with all five score variables as the main exposures to derive the outcome-specific weights and the outcome-specific HLIs. Then, Cox models were constructed by considering, in turn, each version of the HLI as the main exposure. The HLI was consistently modelled in continuous using a linear term on the log-hazard-rate scale.

### Evaluation criteria

#### HR estimates and discriminatory power

 For each event, HR estimates and corresponding 95% confidence intervals (CIs) were computed for a 1-standard deviation (SD) increase of the different versions of the HLI. They allowed the comparison of the estimated association between overall adherence to a healthy lifestyle and the event being studied, depending on the version of the HLI being used. To further illustrate how risk stratification may be hindered when using an unweighted rather than an outcome-specific weighted HLI, we considered the 2^5^ = 32 lifestyle profiles corresponding to each possible combination of the five binary scores *Smoking*^*(0–1)*^, *Alcohol*^*(0–1)*^, *Diet*^*(0–1)*^, *PA*^*(0–1)*^ and *Adipo*^*(0–1)*^. Setting the unhealthiest profile {*Smoking*^*(0–1)*^ = 0, *Alcohol*^*(0–1)*^ = 0, *Diet*^*(0–1)*^ = 0, *PA*^*(0–1)*^ = 0, *Adipo*^*(0–1)*^ = 0} as the reference, we compared the HR estimates for the other 31 profiles produced by Cox models utilizing the standard and outcome-specific HLIs, respectively.

More generally, the discriminatory power of models based on the different versions of the HLI was quantified using Harrell’s C-index. HRs and Harrell’s C-indices were primarily computed in the full EPIC study population. For models based on outcome-specific HLIs, this amounted to evaluating them on the data used for their construction, which could create bias if overfitting was present. Cross-validation was applied to assess this bias: the EPIC study population was randomly split into *(i)* a training sample (75% of the total sample) where the outcome-specific weights were estimated, and *(ii)* a test sample (the remaining 25% of the total sample) where HRs and Harrell’s C-indices were computed. This process was repeated 10 times to prevent possible dependency on a single split^34^. HRs and Harrell’s C-indices were averaged over these 10 repetitions and compared to the values obtained on the total EPIC study population to assess the bias magnitude.

#### Population attributable fractions

 For each specific outcome, we computed PAFs at age *a*, defined as

1 $$PAF\left(a\right)= \frac{P\left(Y<a\right)-P\left({Y}^{\left(max\right)}<a\right)}{P\left(Y<a\right)}$$

Here, $P\left(Y<a\right)$ is the event risk before age *a* in the EPIC study population and $P\left({Y}^{\left(max\right)}<a\right)$ is the hypothetical event risk before age *a* in the counterfactual EPIC study population where, for all participants, all five lifestyle summary variables would have been set to their maximal possible values, while all other variables used for adjustment or stratification would have been set to their actual value observed in EPIC. Under technical conditions^35^, $PAF\left(a\right)$ coincides with the proportion $P\left({Y}^{\left(max\right)}>a| Y<a\right)$ of events before age *a* that would have been prevented had all EPIC participants adhered to the “healthiest” behavior regarding all five lifestyle components. Absolute risks $P\left(Y<a\right)$ and counterfactual absolute risks $P\left({Y}^{\left(max\right)}<a\right)$ were estimated by averaging the individual risk predictions in the EPIC study population, and in the counterfactual populations, respectively. Non-parametric bootstrap based on 100 bootstrapped samples was used to estimate the corresponding 95% CI.

All analyses were performed using the R software, version 4.1.2. Given the nature of the weights used in the definition of the outcome-specific HLIs, models utilizing individual lifestyle scores would achieve similar discriminatory power and produce similar PAF estimates when compared to models based on outcome-specific HLIs.

### Ethics

The EPIC study was conducted according to the Declaration of Helsinki and approved by the ethics committee at the International Agency for Research on Cancer (IARC) on 12 January 1995 and on 10 May 2017 (re-evaluation). Written informed consent was obtained from all subjects involved in the study.

## Results

### Study population

**Figure 1:**
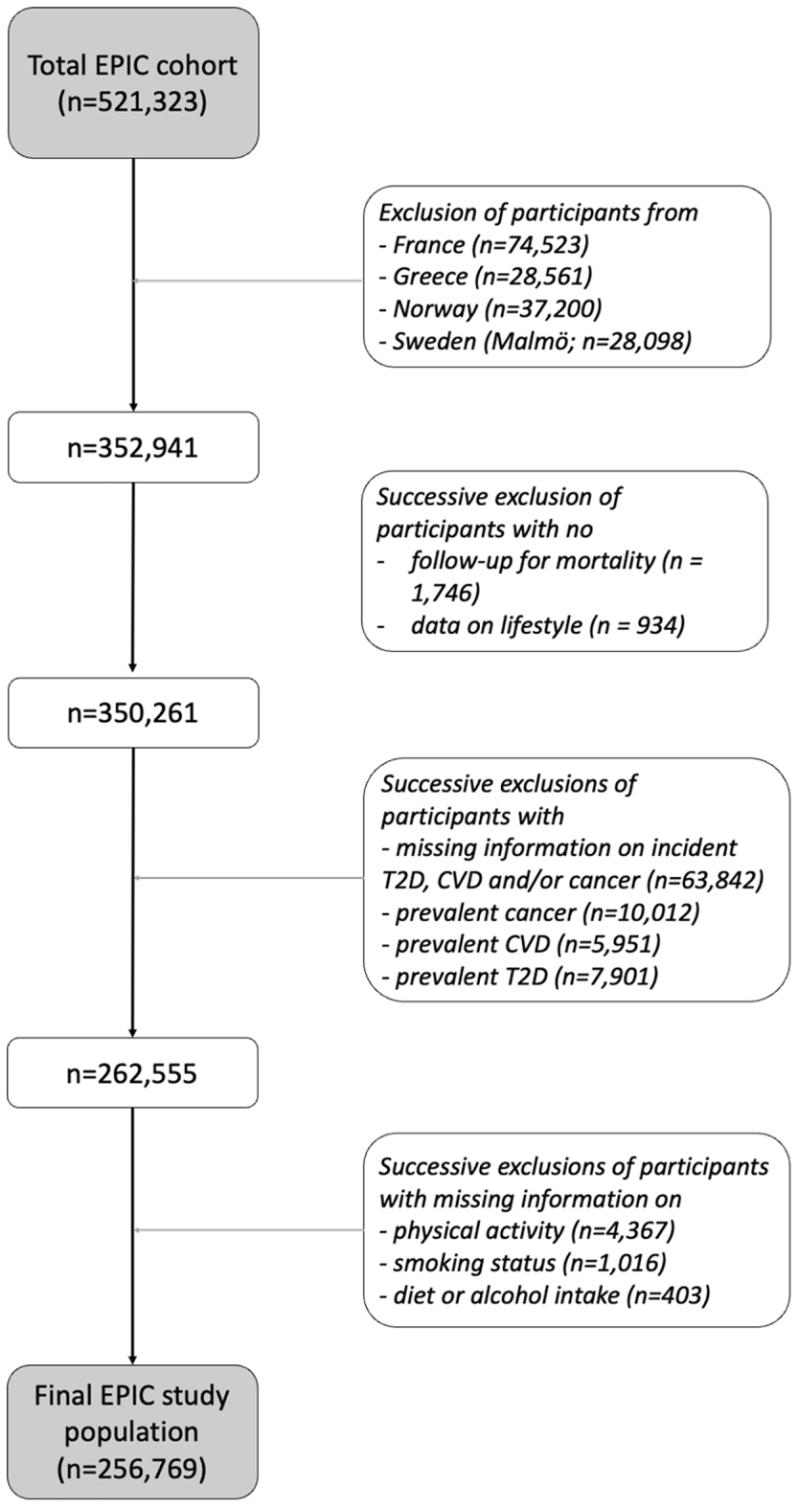
Flowchart summarizing the exclusion criteria that led to the final EPIC study population used in our analyses.

The final study population comprised 256,769 EPIC participants (Fig. 1), including 99,098 men (38.6%) and 157,671 women (61.4%) (Table 2). Average follow-up time and total number of incident events were 16.3 (SD = 3.4) years and 25,191 for all-cause mortality, 10.9 (2.3) years and 11,763 for T2D, 11.5 (2.8) years and 11,766 for CVD, and 14.3 (4.0) years and 34,159 for cancer.

**Table 2:** Main characteristics of the EPIC study population.

	EPIC study population
	(N = 256,769)
Age at recruitment (years)	
Mean (SD)	51.4 (9.33)
Length of follow-up for mortality (years)	
Mean (SD)	16.3 (3.37)
Length of follow-up for T2D (years)	
Mean (SD)	10.9 (2.34)
Length of follow-up for CVD (years)	
Mean (SD)	11.5 (2.78)
Length of follow-up for cancer (years)	
Mean (SD)	14.3 (3.96)
Country	
Italy	43,144 (16.8%)
Spain	35,827 (14.0%)
United Kingdom	29,937 (11.7%)
The Netherlands	29,141 (11.3%)
Germany	42,937 (16.7%)
Sweden	23,523 (9.2%)
Denmark	52,260 (20.4%)
Sex	
Male	99,098 (38.6%)
Female	157,671 (61.4%)
Use of postmenopausal hormone	
Male	99,098 (38.6%)
No	128,478 (50.0%)
Yes	23,045 (9.0%)
Missing	6,148 (2.4%)
Education	
None	12,392 (4.8%)
Primary school completed	78,612 (30.6%)
Technical/professional school	68,795 (26.8%)
Secondary school	39,535 (15.4%)
Longer education (incl. University deg.)	52,991 (20.6%)
Not specified	4,444 (1.7%)
Smoking status	
Never smoker	119,185 (46.4%)
Former smoker	72,015 (28.0%)
Current smoker	65,569 (25.5%)
Alcohol intake (g/day)	
Mean (SD)	13.5 (18.9)
Mediterranean diet score	
Mean (SD)	7.59 (2.96)
Physical activity	
Inactive	53,845 (21.0%)
Moderately inactive	85,003 (33.1%)
Moderately active	59,541 (23.2%)
Active	58,380 (22.7%)
BMI (kg/m^2^)	
Mean (SD)	25.9 (4.13)
Standard HLI	
Mean (SD)	11.2 (3.30)
Simplified standard HLI	
Mean (SD)	3.21 (1.02)

### HLIs based on binary scores

#### Outcome-specific HLIs

 Weights of the main terms used in the definition of the outcome-specific HLIs were all positive, except the one of *Alcohol*^*(0–1)*^ for the T2D- and CVD- specific HLIs (Table 3). Smoking had the strongest impact on the all-cause mortality, CVD- and cancer- specific HLIs, while adiposity had the strongest impact on the T2D-specific HLI. Alcohol had the weakest impact on the all-cause mortality specific HLI, while it was diet for the T2D- and CVD- specific HLI, and physical activity for the cancer-specific HLI. Several interaction terms were selected in the outcome-specific HLIs. For example, *Alcohol*^*(0–1)*^ * Diet^*(0–1)*^ was selected with a negative weight in the simplified death- and CVD-specific HLIs (Table 3).

**Table 3:** Weights used in the construction of the outcome-specific HLI based on binary scores for the 5 individual lifestyle components.

	Death	T2D	CVD	Cancer
Main terms				
Alcohol^*(0–1)*^	0.300	− 0.423	− 0.445	0.342
Adiposity^*(0–1)*^	0.984	2.450	0.900	0.671
Diet^*(0–1)*^	1.030	0.050	0.176	0.975
PA^*(0–1)*^	0.421	0.328	0.214	0.268
Smoking^*(0–1)*^	2.450	0.290	1.940	2.360
Interactions				
Alcohol^*(0–1)*^ * Adiposity^*(0–1)*^		0.291		
Alcohol^*(0–1)*^ * Diet^*(0–1)*^	− 0.291		− 0.255	
Alcohol^*(0–1)*^ * Smoking^*(0–1)*^	− 0.184		0.295	
Adiposity^*(0–1)*^ * Diet^*(0–1)*^	− 0.236			
Diet^*(0–1)*^ * Smoking^*(0–1)*^	− 0.505	0.209		− 0.608
Diet^*(0–1)*^ * PA^*(0–1)*^			0.255	

Weights were derived from Cox models and scaled so that outcome-specific HLIs had unit variance. Cox models used age as the main time scale, were stratified on study center, sex, and age at recruitment, and were adjusted for education level, height, and energy intake from non-alcoholic sources, and, for women, menopausal status and use of postmenopausal hormones.

Supplementary Figure S1 presents the empirical distributions of the outcome-specific HLIs and, for comparison, of the standard HLI (after scaling it to a unit standard deviation). The distribution of the standard HLI was approximately symmetrical and centered around 3, while the distributions of all the outcome-specific HLIs were more skewed, with most values in the top range of the distributions.

#### HRs, risk stratification and discriminatory power

**Figure 2:**
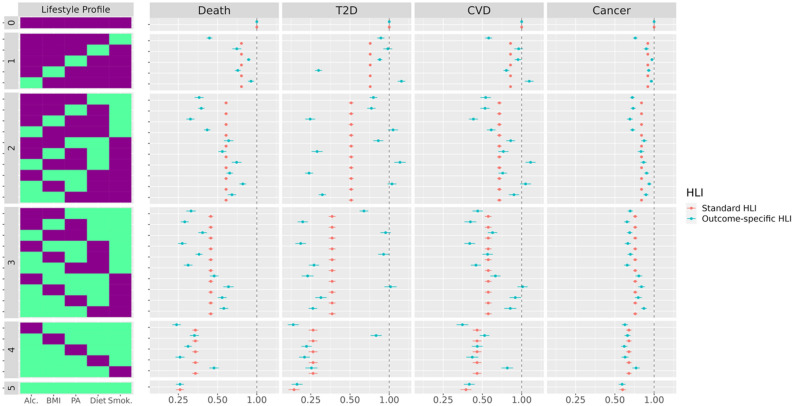
Premature mortlity, T2D, CVD and cancer hazard ratios in relation to the 32 possible lifestyle profiles defined by combining the 5 binary lifestyle scores. The “unhealthiest” profile, corresponding to all five summary variables equal to 0, was set as the reference. The heatmap describes each profile in terms of the 5 lifestyle scores, with purple and green entries corresponding to value 0 (unhealthy behavior) and value 1 (healthy behavior), respectively. Profiles were grouped according to their standard HLI value. Cox models used age as the main time scale, were stratified on study center, sex, and age at recruitment, and were adjusted for education level, height, and energy intake from non-alcoholic sources, and, for women, menopausal status and use of postmenopausal hormones.

 As displayed in Table 4, outcome-specific HLIs were more strongly associated with risk of the corresponding outcome than the standard HLI. For T2D for example, the HR was 0.71 (0.70, 0.72) and 0.60 (0.59, 0.61) per 1-SD increment of the standard and outcome-specific HLI, respectively. HR estimates for the 32 lifestyle profiles corresponding to each possible combination of the five binary scores highlighted that different lifestyle profiles leading to the same value of the standard HLI could be associated with sizably different hazard ratios (Fig. 2). Among all five lifestyle profiles leading to a standard HLI value of 1, the profile {*Smoking*^*(0–1)*^ = 1, *Alcohol*^*(0–1)*^ = 0, *Diet*^*(0–1)*^ = 0, *PA*^*(0–1)*^ = 0, *Adipo*^*(0–1)*^ = 0} had a much lower HR for all-cause mortality, and to a lesser extent, CVD and cancer, compared to the other four profiles, while the profile {*Smoking*^*(0–1)*^ = 0, *Alcohol*^*(0–1)*^ = 0, *Diet*^*(0–1)*^ = 0, *PA*^*(0–1)*^ = 0, *Adipo*^*(0–1)*^ = 1} had the lowest HR for T2D, thus mirroring the respective impacts of the lifestyle components on the different outcome-specific HLIs.

**Table 4:** HR (for a 1-SD increase) and Harrell’s C-index of the standard and outcome-specific HLIs for all-cause mortality, T2D, CVD and cancer.

Event	HR^a^ (1-SD increase)		Harrell’s C-index^b^	
	Standard HLI	Outcome-specific HLI	Standard HLI	Outcome-specific HLI
Simplified HLI (based on binary scores)				
Death	0.76 (0.75, 0.77)	0.71 (0.70, 0.72)	0.60 (0.60, 0.61)	0.62 (0.62, 0.63)
T2D	0.71 (0.70, 0.72)	0.60 (0.59, 0.61)	0.61 (0.60, 0.62)	0.67 (0.66, 0.68)
CVD	0.82 (0.80, 0.83)	0.74 (0.73, 0.75)	0.59 (0.58, 0.60)	0.61 (0.60, 0.62)
Cancer	0.89 (0.88, 0.90)	0.87 (0.86, 0.88)	0.54 (0.54, 0.55)	0.55 (0.54, 0.55)
HLI (based on categorical scores)				
Death	0.73 (0.72, 0.74)	0.68 (0.68, 0.69)	0.61 (0.61, 0.62)	0.64 (0.63, 0.64)
T2D	0.66 (0.64, 0.67)	0.43 (0.42, 0.44)	0.63 (0.62, 0.64)	0.72 (0.72, 0.73)
CVD	0.77 (0.75, 0.78)	0.70 (0.69, 0.72)	0.60 (0.59, 0.61)	0.62 (0.61, 0.63)
Cancer	0.88 (0.87, 0.89)	0.85 (0.84, 0.86)	0.55 (0.54, 0.55)	0.55 (0.55, 0.56)

^**a**^Cox models used age as the main time scale, were stratified on study center, sex, and age at recruitment, and were adjusted for education level, height, and energy intake from non-alcoholic sources, and, for women, menopausal status and use of postmenopausal hormones.

Models based on outcome-specific HLIs achieved a larger discriminatory power compared to those based on the standard HLI. For T2D, Harrell’s C-index was 0.67 (0.66, 0.68) and 0.61 (0.60, 0.62) for the models based on the outcome-specific and standard HLIs, respectively. Cross-validated estimates were similar to those computed on the total EPIC study population (Supplementary Table 2), suggesting low to null bias due to over-fitting in our main analysis.

#### Population attributable fractions

**Figure 3:**
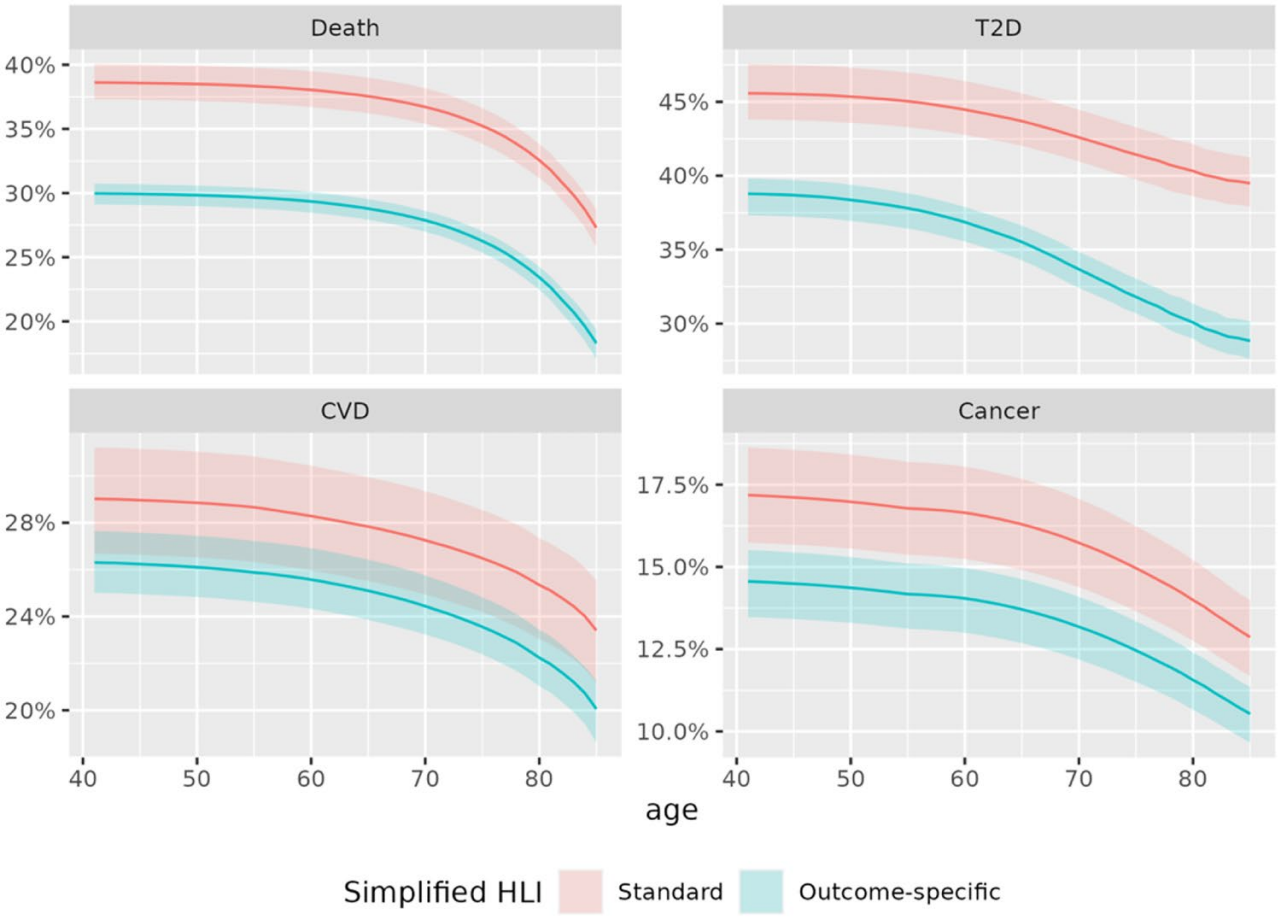
Estimated PAF of unhealthy lifestyle for death, T2D, CVD and Cancer, as a function of age. Estimations were derived from models based on the standard (red) and outcome-specific (cyan) HLIs, using binary scores for each lifestyle component.

 PAFs were consistently larger in analyses based on the standard HLI compared to those based on outcome-specific HLIs. The proportion of deaths by the age of 80 that would have been prevented had the whole population adhered to the “healthiest” lifestyle habits was estimated to be 33% (31%, 34%) and 23% (22%, 24%) when using the standard HLI and the mortality-specific HLI, respectively (Fig. 3). Similar patterns were observed for the other three outcomes (Figs. 3).

### HLIs based on categorical scores

**Figure 4:**
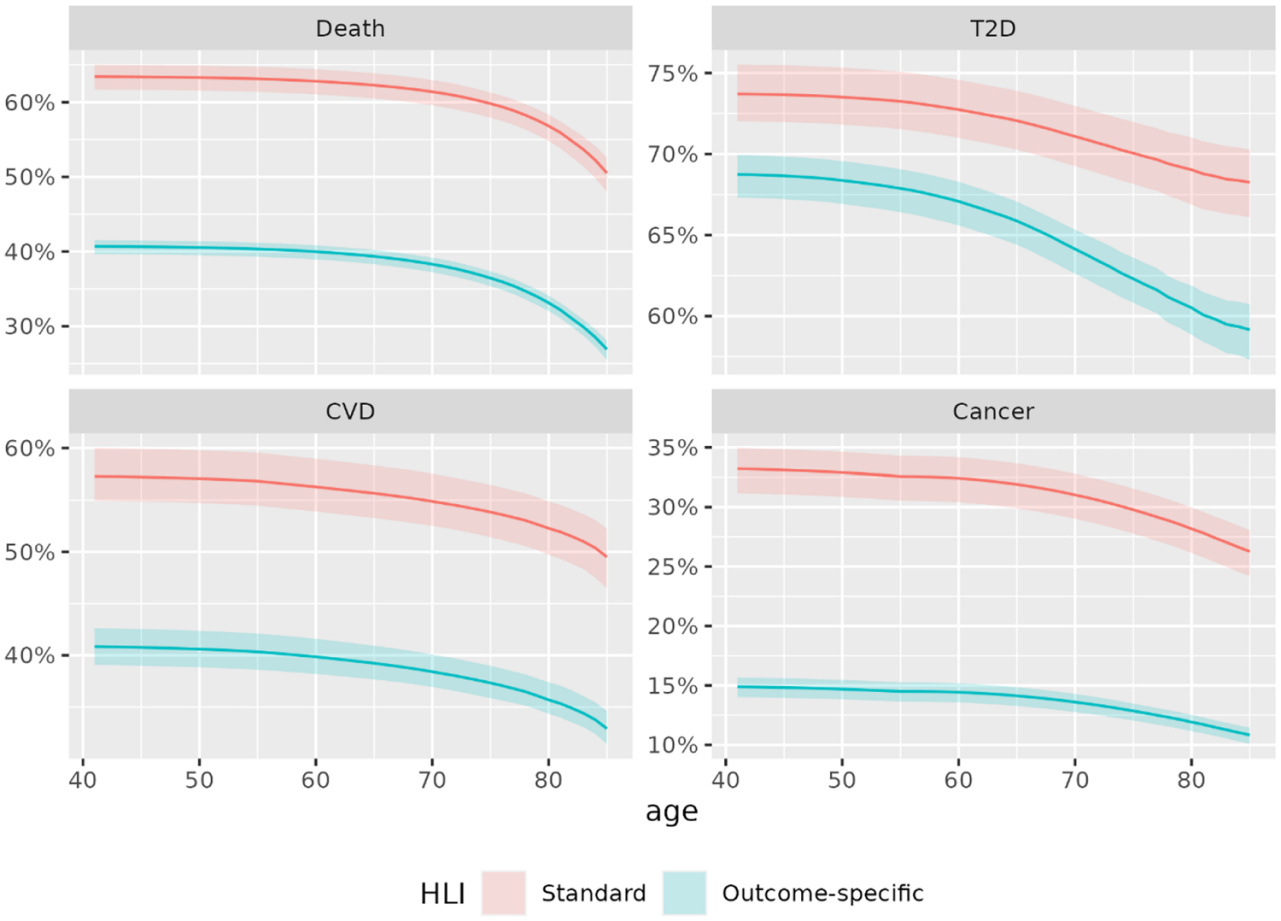
Estimated PAF of unhealthy lifestyle for death, T2D, CVD and cancer, as a function of age. Estimations were derived from models based on the standard (red) and outcome-specific (cyan) HLIs, using categorical scores for each lifestyle component.

Overall, the “healthiest” categories of each individual component received the largest weights in the outcome-specific HLIs (Supplementary Table 2). Consistent with what we observed when using binary scores, smoking had the strongest impact on the all-cause mortality, CVD- and cancer- specific HLIs, while adiposity had the strongest impact on the T2D-specific HLI. The distribution of the standard HLI was approximately symmetrical around its mean value (Supplementary Fig. 2). The distributions of the all-cause mortality-, CVD- and cancer-specific HLIs were more skewed, with most values in the top range of the distributions, while the T2D-specific HLI had a multi-modal distribution. Patterns of differences in HR, C-index and PAF estimated from analyses based on standard and outcome-specific HLIs were similar, yet more pronounced, when utilizing categorical scores compared to the simplified setting of binary scores presented above. For T2D, the HR was 0.66 (0.64, 0.67) and 0.43 (0.42, 0.44) per 1-SD increment of the standard and outcome-specific HLIs, respectively, while the corresponding C-index were 0.63 (0.62, 0.64) and 0.72(0.72, 0.73). The proportion of deaths by the age of 80 that would have been prevented had the whole population adhered to the “healthiest” lifestyle habits was estimated to be 57% (55%, 58%) and 33% (32%, 34%) when considering the standard and death-specific HLI, respectively (Fig. 4).

### Theoretical study of linear causal models

Our theoretical study under a linear causal model presented in the Supplementary Material showed that the regression parameter of the scaled weighted composite score was always larger than that of the scaled unweighted composite score, unless the weighted and unweighted composite scores coincided. Considering the analog of the PAF, we showed that analyses utilizing the unweighted composite score yielded either downward or upward biased estimates, while analyses based on the weighted composite score yielded unbiased estimates.

## Discussion

In this study, we introduced a novel HLI to account for the magnitude of the relationships between individual lifestyle components and specific disease outcomes, using data-driven weights. The standard and the outcome-specific versions of the HLI were extensively compared by estimating the HR, the C-index and the PAF in a range of scenarios, involving the risk of cancer, T2D, CVD and premature mortality. Two strategies to operationalize the HLI were also investigated, involving in turn, binary indicators or categorical scores for the five components.

In our study, the discriminatory power was consistently larger for models based on the outcome-specific HLI than the standard HLI, sometimes to a large extent as in the case of T2D. The reason for this limitation of the standard HLI was clearly illustrated in Fig. 2, when considering binary indicators. As the standard HLI assumes that all lifestyle components are equally associated with the risk of disease, different lifestyle patterns with the same number of unhealthy components necessarily lead to the same predicted disease hazard rate in analyses based on the standard HLI. Conversely, our analyses utilizing the outcome-specific HLI reflected the disease hazard rate heterogeneity across these lifestyle patterns with the same number of unhealthy components. This limitation of the standard HLI in terms of discriminatory power highlights that it might be a suboptimal analytical choice for risk stratification and/or risk prediction^36^, especially in situations where a given lifestyle component is strongly linked to the outcome under consideration, such as BMI in the case of T2D.

Most previous studies on the HLI used the standard HLI to address etiological questions, specifically to estimate disease-specific HRs to quantify the impact of adhering to healthy lifestyle habits, and disease-specific PAFs to measure the public health burden attributable to unhealthy lifestyles^8,18,19,22,24^. In our study, we observed consistently weaker HR estimates for the standard HLI than the outcome-specific HLI, sometimes to a large extent, as for T2D. These results suggest that analyses utilizing outcome-specific HLIs are more likely to detect associations, particularly for diseases weakly associated with lifestyle habits. Conversely, PAF estimates were consistently larger when using the standard HLI. Estimating weaker HRs and larger PAFs with the standard HLI than the outcome-specific HLI may seem paradoxical, however our results from the theoretical study of linear causal models and the inspection of the empirical distributions of the standard and the outcome-specific HLIs displayed in Figure S1 might help clarify this apparent paradox. According to the binary version of standard HLI, 59% of the EPIC study population had a standard HLI lower or equal to 3 units, i.e., more than 2 standard-deviations below the maximum HLI of 5 units. As a result, the health benefits for this large proportion of participants, had they adhered to the healthiest possible lifestyle, led to large PAF estimates. On the other hand, according to the, say, death-specific HLI, 65% of the study population had an HLI value within one standard-deviation of the maximum HLI. As a result, the benefit in premature mortality had they adhered to the healthiest possible lifestyle was less remarkable, thus explaining the lower PAF estimates. In essence, analyses of the outcome-specific HLI mimics closely an analytical strategy where individual lifestyle components are evaluated jointly within the same model, and therefore yield similar PAF estimates. Thus, our results highlight that analyses based on standard HLI could lead to biased assessments of the public health burden attributable to unhealthy lifestyle. As mentioned in our theoretical study of linear causal models, it could be argued that utilizing standard HLIs might produce approximately valid estimates of PAFs of a latent variable, e.g., reflecting health-consciousness. Yet, the validity of this approach, particularly whether the standard HLI is a better proxy than weighted HLIs for this latent variable, would need further assessment.

The etiology of chronic diseases is complex, and some level of simplification via summary quantities is welcome in epidemiological research. To paraphrase Box’s aphorism, “all summarizations are wrong but some are useful”^37^. To be useful, a summarization should produce approximately valid results. The validity of results in analyses based on the standard HLI could be assessed by comparing them to results of an outcome-specific HLI or the individual lifestyle components. If results are similar, the standard HLI could be appropriate as it does not rely on data-driven weights and it could facilitate the comparison of findings across studies and across health-related outcomes. However, the premise that standard HLIs would facilitate comparison across studies might be tempered in view of the myriad of versions of standard HLIs proposed in the literature^5,6,9,10,17,18,38^.

Multiple lifestyle factors influence an individual’s health, but some are more critical than others, which should be reflected in public health recommendations. Towards this aim, the “healthiest” lifestyle profiles could be defined as the combinations of individual lifestyle behaviors associated with lowest risk of disease, longest life expectancy, or longest life expectancy free of a chronic disease^9^. The development and validation of an HLI using weights derived from meta-analyzed associations with disease risk, mortality or a composite outcome reflecting mortality and common chronic diseases would help the characterization and promotion of these healthiest profiles.

In line with previous versions of the standard HLI^8^, the HLI considered in this study was based on five individual components: smoking habits, alcohol intake, diet, physical activity, and adiposity. Refined statistical methods, e.g. using splines, could be used to combine the individual components into an outcome-specific HLI. Also, working with a refined categorization of these five components, including more descriptors, such as more refined information on smoking intensity or adiposity or a broader spectrum of dietary exposures, and/or including information on other lifestyle factors, such as quality of sleep^39,40^ or stress, might lead to more accurate assessments of the relationship between lifestyle and health-related outcomes. The evaluation conducted in this study relied on the EPIC cohort, where the study populations in the various countries were generally more health-conscious than their source populations. We could not account for other major chronic diseases that could affect observed associations with our outcomes of interest because of a lack of such information in EPIC. For example, chronic obstructive pulmonary disease (COPD) frequently co-occurs with CVD and share tobacco smoking as a main risk factor^41^. These potential limitations were acknowledged, yet they were unlikely to affect the main conclusions of the study, which were corroborated by the evidence of our theoretical results under simple linear causal models.

## Conclusions

The assessment of the relationship between a holistic composite score reflecting adherence to healthy lifestyle behaviors and the risk of disease and mortality could be improved by utilizing outcome-specific versions of the HLI. Standard HLIs can lead to biased assessments of the public health burden attributable to unhealthy lifestyles. The development and validation of data-driven HLIs best predicting the occurrence of disease outcomes is instrumental for the assessment of lifestyle and disease risk associations and the generation of accurate public health recommendations.

### Supplementary Information

Supplementary Information.

## Data Availability

The EPIC data is not publicly available, but access requests can be submitted to the Steering Committee (https://epic.iarc.fr/access/index.php).
