# Supplementary material for: On the use of the healthy lifestyle index to investigate specific disease outcomes
Source: Sci Rep. 2024 Jul 15;14:16330. doi: 10.1038/s41598-024-66772-w (PMC11250810; doi:10.1038/s41598-024-66772-w)
Supplement: Supplementary file 1 — Supplementary Information. [file 41598_2024_66772_MOESM1_ESM.docx]

**Supplementary Materials**

**On the use of the healthy lifestyle index to investigate specific disease outcomes**

Vivian Viallon^*1^, et al.

*Corresponding author: [viallonv@iarc.who.int](mailto:viallonv@iarc.who.int)

^1^International Agency for Research on Cancer (IARC-WHO), Lyon, France

**Supplementary Tables and Figures**

**Table S1:** Weights of the outcome-specific HLI based on categorical scores for each lifestyle component. For simplicity, weights provided here were those derived from models^1^ focusing on main effects (*i.e.*, ignoring interactions).

| Lifestyle Component | Category | Death | T2D | CVD | Cancer |
| --- | --- | --- | --- | --- | --- |
| Alcohol intake | Alcohol intake ≥60 g/day | 0 | 0 | 0 | 0 |
|  | Alcohol intake ≥25 to <60g/day | 1.04 | 0.145 | 0.086 | 0.888 |
|  | Alcohol intake ≥12 to <25 g/day | 1.30 | 0.190 | 0.219 | 1.030 |
|  | Alcohol intake ≥6 to <12 g/day | 1.41 | 0.193 | 0.018 | 1.360 |
|  | Alcohol intake <6g/day) | 1.06 | 0.001 | 0.308 | 1.250 |
| Adiposity | BMI ≥30 kg/m^2^ | 0 | 0 | 0 | 0 |
|  | BMI ≥26 to <30 kg/m^2^ | 0.638 | 1.090 | 0.416 | 0.509 |
|  | BMI ≥24 to <26kg/m^2^ | 0.824 | 1.940 | 0.856 | 0.675 |
|  | BMI <22 kg/m^2^ | 0.295 | 3.010 | 1.400 | 0.551 |
|  | BMI ≥22 to <24 kg/m^2^ | 0.722 | 2.520 | 1.190 | 0.673 |
| Diet | 1^st^ quintile Med. Diet score | 0 | 0 | 0 | 0 |
|  | 2^nd^ quintile Med. Diet score | 0.254 | 0.088 | 0.157 | 0.103 |
|  | 3^rd^ quintile Med. Diet score | 0.321 | 0.127 | 0.176 | 0.419 |
|  | 4^th^ quintile Med. Diet score | 0.454 | 0.146 | 0.151 | 0.600 |
|  | 5^th^ quintile Med. Diet score | 0.573 | 0.142 | 0.360 | 0.746 |
| Physical activity | Physically inactive | 0 | 0 | 0 | 0 |
|  | Physically moderately inactive | 0.449 | 0.150 | 0.294 | 0.355 |
|  | Physically moderately active | 0.589 | 0.245 | 0.429 | 0.406 |
|  | Physically active | 0.725 | 0.299 | 0.489 | 0.539 |
| Smoking | Current smoker | 0 | 0 | 0 | 0 |
|  | Former smoker | 1.540 | 0.250 | 1.530 | 1.480 |
|  | Never smoker | 2.040 | 0.381 | 2.110 | 2.010 |

**^1^**Cox models used age as the main time scale, were stratified on study center, sex, and age at recruitment, and were adjusted for education level, height, and energy intake from non-alcoholic sources, and, for women, menopausal status and use of postmenopausal hormones

**Table S2:** Comparison between estimates derived on the full EPIC study population and cross-validated estimates, for the HR (for a 1-SD increase) and Harrell’s C-index.

| Event | HR^1^ (1SD increase) | | | Harrell’s C-index^1^ | |
| --- | --- | --- | --- | --- | --- |
|  | **Standard HLI** | **Outcome-specific HLI** | | **Standard HLI** | **Outcome-specific HLI** |
|  | *Simplified HLI (based on binary scores)* | | | | |
| Death | 0.759 -> 0.761 | | 0.711 -> 0.710 | 0.604 -> 0.604 | 0.625 -> 0.627 |
| T2D | 0.711 -> 0.709 | | 0.602 -> 0.602 | 0.630 -> 0.633 | 0.670 -> 0.668 |
| CVD | 0.819 -> 0.817 | | 0.741 -> 0.740 | 0.589 -> 0.588 | 0.613 -> 0.610 |
| Cancer | 0.893 -> 0.889 | | 0.869 -> 0.866 | 0.542 -> 0.544 | 0.548 -> 0.549 |
|  | *HLI (based on categorical scores)* | | | | |
| Death | 0.727 -> 0.729 | | 0.682 -> 0.688 | 0.612 -> 0.612 | 0.638 -> 0.635 |
| T2D | 0.657 -> 0.654 | | 0.426 -> 0.426 | 0.617 -> 0.617 | 0.722 -> 0.723 |
| CVD | 0.765 -> 0.755 | | 0.702 -> 0.703 | 0.600 -> 0.603 | 0.620 -> 0.620 |
| Cancer | 0.876 -> 0.873 | | 0.851 -> 0.859 | 0.546 -> 0.547 | 0.554 -> 0.552 |

^1^Cox models used age as the main time scale, were stratified on study center, sex, and age at recruitment, and were adjusted for education level, height, and energy intake from non-alcoholic sources, and, for women, menopausal status and use of postmenopausal hormones

**Figure S1:** Distributions of the standard HLI and the outcome-specific HLIs for all-cause mortality, T2D, CVD and cancer in the EPIC population study, using binary lifestyle scores. The standard HLI was scaled so that its standard deviation was 1 in the EPIC population study; the standard deviations of the outcome-specific HLIs were 1 by construction.

**
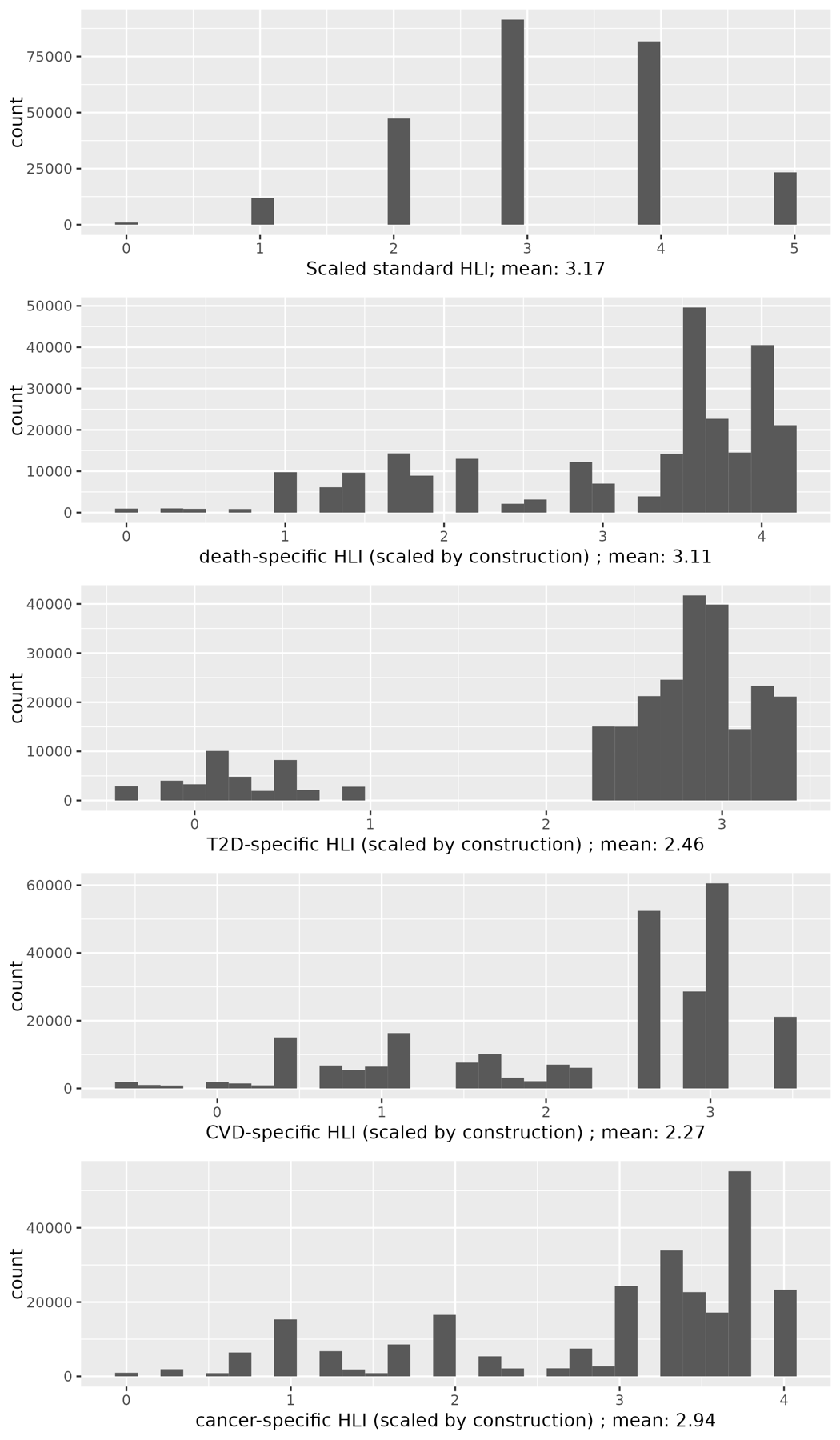
**

**Figure S2:** Distributions of the standard HLI and the outcome-specific HLIs for all-cause mortality, T2D, CVD and cancer in the EPIC population study, using categorical lifestyle scores. The standard HLI was scaled so that its standard deviation was 1 in the EPIC population study; the standard deviations of the outcome-specific HLIs were 1 by construction.

**
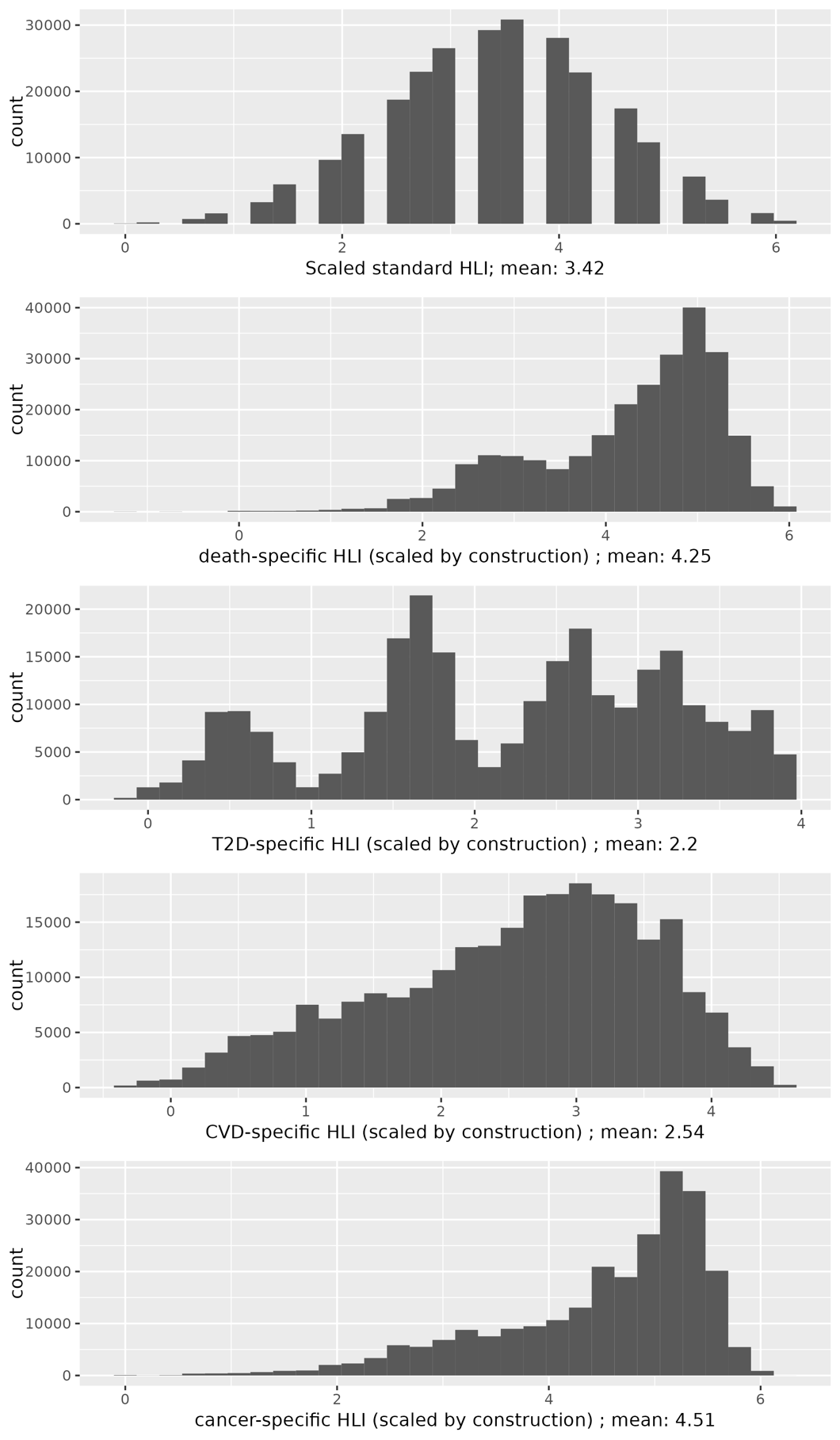
**

**Theoretical study under a simple linear causal model**

Let $X_{1}$, …, $X_{p}$ denote *p* real-valued variables, for some *p > 1*, which are assumed to be causally related to the outcome of interest $Y$ under the following causal structural model[35],

$Y = \sum_{j=1}^{p} {\beta_{j}X}_{j}+ \varepsilon.$ (1)

Here, $Y$ and $\varepsilon$ are real-valued random variables, and $\varepsilon$ is assumed to be centered. For all *j*, we will assume that $\beta_{j}$ is non-positive, the possible values of $X_{j}$ lie in the interval $\left[ 0, \tau\right]$ for some $\tau$ > 0, and sd($X_{j}$) = $\sigma$ where sd denotes standard deviation.

*Graphical representation of the causal model and integration of composite scores*

Consider the weighted and unweighted composite scores $\sum_{j=1}^{p} {\beta_{j}X}_{j}$ and $\sum_{j=1}^{p} X_{j}$. Further denote their scaled version by $Z^{*}$and $Z$, respectively, so that sd($Z^{*}$) = sd($Z$) = 1. Under the causal model (1), represented graphically on Figure S.2, the variables $X_{1}$, …, $X_{p}$ influence the outcome variable $Y$ through $Z^{*}$only. They also (deterministically) influence the unweighted composite score $Z$, which is usually not a cause of $Y$. It is a cause of $Y$ in some cases, including when $Z=Z^{*}$(left panel of Figure S.3), or when $Z$ can be regarded as (a good proxy for) a possibly latent variable that is a cause of $Y$, and whose effect on $Y$ is entirely mediated by $X_{1}$, …, $X_{p}$ (right panel of Figure S.3).

*Regression parameters under linear models based on composite scores*

Consider the following two working linear regression models

$Y = {\alpha^{*}+ \gamma}^{*}Z^{*}+ \varepsilon$ (2)

$Y = \alpha+ \gamma Z+ \epsilon$ (3)

Denote by $\beta$ the vector of parameters ($\beta_{j}, \ldots, \beta_{j}$)^T^, and by $\left\| . \right\|_{2}$ and $\left\| . \right\|_{1}$ the *L_2_-*and *L_1_-*norm of vectors, respectively. Under the causal model (1), $\alpha^{*}=0, {\alpha= \sum_{j=1}^{p} {(\beta}_{j}- \gamma)E_{j}, \gamma}^{*}=$ $\sigma\left\| \beta\right\|_{2}$, and $\gamma$ = $\sigma{\left\| \beta\right\|_{1}p}^{-1/2}$. Then, because $\left\| v \right\|_{1}\leq{\sqrt{p} \left\| v \right\|}_{2}$for any *p*-vector $v$ , it follows that

$\gamma\leq\gamma^{*}$ (4)

with equality if and only if the $\beta_{j}$’s are all equal. In other words, the regression parameter of the (scaled) unweighted composite score $Z$ is lower than that of the (scaled) weighted composite score $Z^{*}$, unless the $\beta_{j}$’s are all equal (in which case ${Z= Z}^{*}$), which is consistent with what we observed in our empirical comparison under Cox models.

*Counterfactual quantities*

Denote by $Y^{(max)}$ the counterfactual outcome variable that would have been observed in the world following the intervention *do*($X_{j}$= $\tau$) for all *j*[35]. For any random variable *U,* denote its expectation by $E$*U.* The counterfactual quantity $C^{(max)}= E\left( Y- Y^{(max)} \right)$ represents the difference between the expected values of the outcome in the actual world and in the counterfactual world following the intervention *do*($X_{j}$= $\tau$) for all *j*, respectively. It can be seen as the analog of the PAF we considered in our empirical comparison under Cox models.

In this theoretical analysis, we further consider an additional counterfactual quantity. Denote by $Y^{(0)}$ the counterfactual outcome variable that would have been observed in the world following the intervention *do(*$X_{j}$*=0)* for all *j*, and set $C^{(0)}= E\left( Y^{(0)}- Y \right)$, which is an analog to the following counterfactual quantity in a survival setting

$$\frac{P\left( Y^{\left( 0 \right)}<a \right)- P\left( Y<a \right)}{P\left( Y<a \right)}$$

Expressions of $C^{(max)}$ and $C^{(0)}$ can be derived under model (1) or, equivalently, under model (2). Denoting by $E_{j}$ the expectation of $X_{j}$, it follows from simple algebra that

$C^{(max)}= \sum_{j=1}^{p} \beta_{j}(\tau- E_{j} )$ and $C^{(0)}= \sum_{j=1}^{p} \beta_{j}E_{j}$.

On the other hand, deriving counterfactual quantities under model (3) amounts to considering that the underlying causal model is that of Figure S.3, which is usually mis-specified if the true underlying causal model is model (1). Denote by $\tilde{C}^{(max)}$ and $\tilde{C}^{(0)}$the versions of $C^{(max)}$ and $C^{(0)}$derived when (generally erroneously) considering model (3) as the underlying causal model. Further set $\bar{\beta}=\sum_{j=1}^{p} \beta_{j}/p$. Then, it can be shown that $\tilde{C}^{(max)} = \bar{\beta}\sum_{j=1}^{p} (\tau- E_{j} )$ and $\tilde{C}^{(0)}= \bar{\beta}\sum_{j=1}^{p} E_{j}$.

Consequently, we have

$$\Delta^{(max)}= {\tilde{C}^{(max)}- C}^{(max)}= -\sum_{j=1}^{p} {(\beta}_{j}-\bar{\beta}) E_{j}$$

and

${\Delta^{(0)}=\tilde{C}}^{(0)}- C^{\left( 0 \right)}= \sum_{j=1}^{p} {(\beta}_{j}-\bar{\beta}) E_{j}= -\Delta^{(max)}$.

Both $\Delta^{(max)}$ and $\Delta^{(0)}$ are null if the $\beta_{j}$’s are all equal or if the $E_{j}$’s are all equal. However, $\Delta^{(max)}$ and $\Delta^{(0)}$ are generally non-null, they can be either positive or negative, and

$$\Delta^{(max)}>0 \Longleftrightarrow\Delta^{\left( 0 \right)}<0.$$

In other words, counterfactual quantities, such as $C^{(max)}$, derived under model (3) are generally biased, either upward or downward.


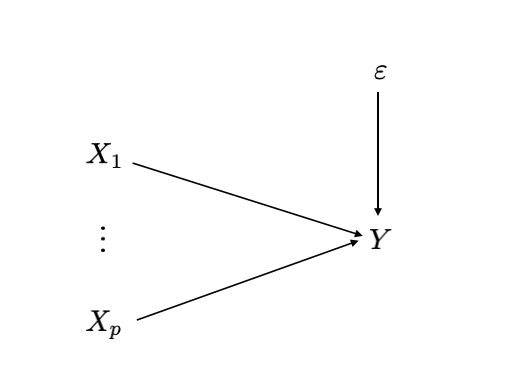

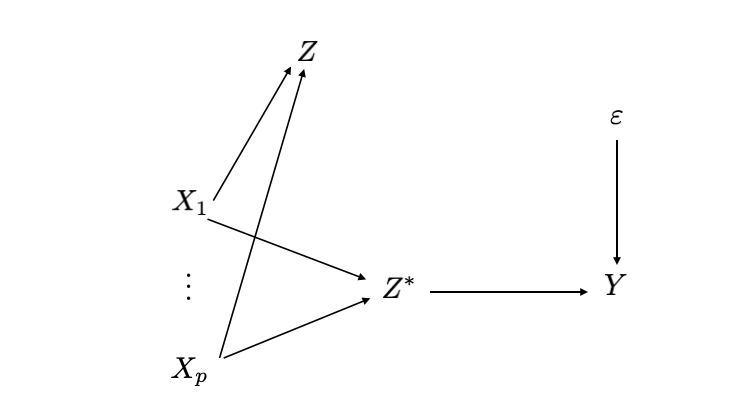


**Figure S.2:** (*Left*) Graphical representation of the causal model (1). (*Right*) The place and role of the weighted and unweighted composite scores under that model.


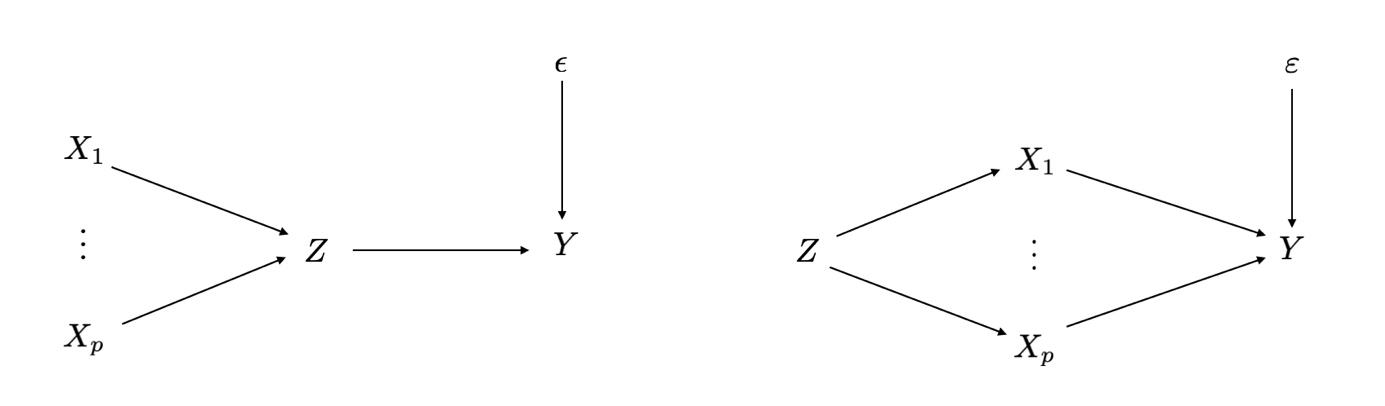


**Figure S.3:** Examples of causal model that are implicitly assumed when deriving counterfactual quantities under the linear regression model (3). *(Left)* The individual variables $X_{j}$ influence the outcome through Z only: under model (1), this means $Z=Z^{*}.$*(Right)* $Z$ corresponds to a latent variable (e.g., health-consciousness) that is the underlying cause of the individual variables $X_{j}.$
